# Unveiling the IL-1β/CXCL2 axis: a shared therapeutic target in periodontitis and inflammatory bowel disease

**DOI:** 10.3389/fimmu.2025.1728773

**Published:** 2026-01-15

**Authors:** Zhongyi Gu, Aichao Gao, Xiang Ma, Xiaotong Wang, Yi Zhang, Yucun Wang, Caiqing Qiu

**Affiliations:** 1 Department of Periodontology, The Affiliated Yantai Stomatological Hospital, Binzhou Medical University, Yantai, China; 2 Department of Gastroenterology, The Affiliated Yantaishan Hospital, Binzhou Medical University, Yantai, China

**Keywords:** chemokines, interleukin-1 beta, inflammatory bowel disease, myeloid cells, oral-gut axis, periodontitis, single-cell RNA sequencing

## Abstract

**Background:**

Periodontitis is clinically associated with inflammatory bowel disease (IBD), yet the shared cellular and molecular programs underpinning this oral–gut inflammatory link remain incompletely defined.

**Methods:**

We integrated bulk gingival transcriptomes from two periodontitis cohorts (GSE16134 and GSE10334) with single-cell RNA-seq data from IBD patients (51,322 cells, 18 samples). After standard quality control and batch-aware integration, we performed pseudobulk differential expression gene (DEG) analysis at the patient level to identify IBD-associated genes and intersected these with periodontitis-consensus DEGs to derive shared signatures. Cell-cell communication networks were inferred using CellChat, and gene set enrichment analysis were conducted to delineated inflammatory signaling. Myeloid subpopulations further resolved to characterize disease-associated functional states. Key findings were assessed in a dual-inflammation rodent model by immunohistochemistry.

**Results:**

We identified 188 common DEGs across the two periodontitis cohorts and 66 genes shared in IBD, which were predominantly enriched in immune compartments and motivated a focused analysis of myeloid cells. Communication analysis revealed extensive network remodeling in IBD, with myeloid populations acting as major signaling hubs. Myeloid subclustering highlighted inflammatory and chemokine-related states characterized by high *IL1B* expression. Among chemokines, *CXCL2* was prioritized because it showed consistent upregulation across bulk periodontitis transcriptomes and IBD myeloid states, and it aligned with prominent IL-1β–chemokine signaling routes inferred from intercellular communication. *In vivo*, IL-1β and CXCL2 signals were increased in the dual-inflammation model, supporting cross-disease consistency of this axis.

**Conclusions:**

Our integrative analyses identify a shared, myeloid-centered IL-1β/chemokine inflammatory program across periodontitis and IBD, which may contribute to the oral–gut inflammatory axis. The IL1β–CXCL2 pathway represents a potentially targetable signaling module, although functional blockade studies are required to establish therapeutic causality.

## 1 Introduction

Periodontitis is a prevalent oral disease characterized by chronic inflammation and progressive destruction of periodontal tissues resulting from dental plaque microbial biofilms, constituting a leading cause of tooth loss in adults worldwide (1, 2). Recent advances in understanding the intricate relationship between oral and systemic health have demonstrated that periodontitis extends beyond localized oral manifestations, potentially contributing to various systemic diseases through mechanisms involving systemic inflammatory burden and immune dysregulation (3, 4). This association is particularly pronounced with chronic inflammatory conditions, including gastrointestinal disorders, cardiovascular diseases, and diabetes mellitus (5). The bidirectional interactions between periodontitis and systemic diseases establish a complex, interconnected inflammatory network with far-reaching clinical implications. Within this framework, the potential association between periodontitis and inflammatory bowel disease (IBD) has emerged as an area of increasing scientific interest and investigation (6, 7).

IBD comprises a group of immune-mediated disorders characterized by chronic gastrointestinal inflammation, encompassing two principal subtypes: ulcerative colitis (UC) and Crohn’s disease (CD) (8). The global incidence of IBD continues to rise annually, profoundly compromising patients’ quality of life while imposing substantial economic burdens on healthcare systems worldwide (9). Although the precise etiology and pathogenesis of IBD remain incompletely understood, accumulating evidence indicates that genetic predisposition, environmental triggers, immune system dysfunction, and intestinal microbiome dysbiosis collectively contribute to disease initiation and progression (10).

Emerging evidence suggests that periodontal pathogens and their metabolites can translocate to the intestine via the oral-gut axis, disrupting intestinal microbial homeostasis, compromising mucosal barrier integrity, and triggering systemic inflammatory cascades (11, 12). Recent clinical epidemiological studies have demonstrated significantly elevated IBD risk among periodontitis patients, indicating potential shared pathological mechanisms (13, 14). However, current understanding of the molecular-level connections between these diseases remains limited, creating a critical knowledge gap that requires comprehensive mechanistic investigation.

Myeloid cells, encompassing monocyte-macrophages, dendritic cells, and neutrophils, represent crucial components of innate immune defense systems and play pivotal roles in inflammatory responses, immune regulation, and tissue repair (15, 16). Accumulating evidence indicates that dysregulated myeloid-cell responses play a central role in the pathogenesis of both periodontitis and IBD by promoting excessive production of pro-inflammatory mediators, including TNF-α, IL-6, IL-1β, and various chemokines (17–19). These inflammatory cascades can perpetuate tissue injury and influence disease progression; however, the specific myeloid cell-mediated pathways that connect periodontitis and IBD remain poorly defined.

The advent of high-throughput sequencing technologies has greatly expanded our ability to study disease mechanisms at the molecular level. Single-cell RNA sequencing enables high-resolution dissection of cellular heterogeneity within disease microenvironments and the identification of cell type–resolved transcriptional patterns (20, 21). In this study, we integrated transcriptomic data from periodontitis and healthy periodontal tissues with single-cell transcriptomic data from IBD patients. This approach enabled the identification of shared inflammatory programs between periodontitis and IBD at the cellular level, with a data-driven focus on myeloid cell–centered pathways. The results may help clarify the molecular basis of the oral–gut inflammatory axis and highlight potentially targetable signaling modules for future clinical translation.

## 2 Materials and methods

### 2.1 Differential gene analysis and functional enrichment in periodontitis

Two periodontitis cohort datasets (GSE16134 (22, 23) and GSE10334 (24)) were downloaded from the Gene Expression Omnibus (GEO) database for comparative analysis (25). GSE16134 (“Bacterial Correlates of Gingival Gene Expression”) contains expression profiling data from 120 patients undergoing periodontal surgery, with a total of 310 interproximal gingival papillae samples (2–4 samples per patient) collected from maxillary posterior regions. This dataset focuses on investigating associations between subgingival bacterial profiles and gene expression patterns in gingival tissues of periodontitis patients. GSE10334 (“Transcriptomes in Healthy and Diseased Gingival Tissues”) comprises transcriptomic data from 90 non-smoking patients (63 with chronic periodontitis and 27 with aggressive periodontitis), generating 247 samples from 183 diseased and 64 healthy gingival sites. Diseased sites were characterized by bleeding on probing, probing pocket depth ≥4mm, and clinical attachment loss ≥3mm, while healthy sites showed no bleeding on probing, probing pocket depth ≤4mm, and clinical attachment loss ≤2mm.

For each cohort, the processed series matrix files were used as the input expression matrices. Probe identifiers were mapped to official gene symbols using the corresponding platform annotation. When multiple probes mapped to the same gene symbol, a single representative probe was retained by selecting the probe with the highest mean expression across all samples. Differential expression analysis between diseased and healthy tissues was performed using the limma package (26). Differentially expressed genes (DEGs) were identified with criteria of adjusted p-value < 0.05 and |log2FC| > 1 (27). DEGs identified from the two cohorts were intersected to derive a consensus periodontitis signature, and concordant direction of change across datasets was verified. A comprehensive protein-protein interaction (PPI) network was constructed using the STRING database (28). Gene Ontology (GO) functional annotation (biological process, BP; molecular function, MF; cellular component, CC) and Kyoto Encyclopedia of Genes and Genomes (KEGG) pathway enrichment analyses were conducted for the common DEGs, with FDR < 0.05 considered statistically significant (29, 30).

### 2.2 Overview of single-cell transcriptomic data in IBD

The IBD single-cell transcriptomic dataset GSE214695 (31) was obtained from the GEO database, encompassing single-cell RNA sequencing data from 6 healthy controls (HC), 6 UC patients, and 6 CD patients. Single-cell data objects were constructed and quality control was performed using the standardized “Seurat” workflow (32). Quality control parameters were set as follows: feature count ranging from 100 to 9000, UMI count ranging from 200 to 50000, retaining genes expressed in at least 4 cells. Cell quality filtering criteria included mitochondrial gene percentage < 50%, ribosomal gene percentage > 3%, and hemoglobin gene percentage < 3% (33). Data were normalized using LogNormalize (scale factor = 10,000), and 2,000 highly variable genes were selected using the “vst” method. Principal component analysis (PCA) was conducted based on the selected variable features. Batch effects across samples were corrected using Harmony with “orig.ident” as the grouping variable (max.iter.harmony = 40). Unsupervised clustering was then performed on the batch-corrected embeddings using 10 principal components and a resolution of 0.05. Cell populations were annotated based on canonical marker genes, including: plasma cells (*JCHAIN, IGKC, IGHA1*), T/NK cells (*CD2, CD3D, NKG7*), epithelial cells (*KRT8, KRT18, TFF3*), myeloid cells (*LYZ, C1QA, IL1B*), fibroblasts (*COL3A1, COL1A1, DCN*), B cells (*CD79A, MS4A1, CD83*), mast cells (*TPSB2, CPA3, GATA2*), and endothelial cells (*IGFBP7, PECAM1, VWF*) (34, 35). Differences in cell-type proportions among HC, UC, and CD groups were assessed at the sample level. Pseudobulk differential expression analysis was performed by aggregating raw UMI counts at the sample level (36). Specifically, the raw count matrix was extracted from the Seurat object (“counts” slot), and cells were grouped by a composite identifier combining sample origin and disease group (aggregate_id = orig.ident + groups). For each aggregate_id, gene-level counts were summed across all cells using Matrix::rowSums to generate a pseudobulk count matrix. A corresponding sample annotation table was constructed containing the disease condition for each pseudobulk profile. Differential expression analysis was conducted using DESeq2 with the design formula ~ condition, where normalization for library size was performed internally via DESeq2 size-factor estimation. Genes with adjusted p-value < 0.05 and |log2FC| > 1 were defined as IBD-associated DEGs (37). Shared DEGs were defined as the intersection between IBD pseudobulk DEGs and periodontitis DEGs, and their expression patterns across annotated cell types were visualized using heatmaps and dot plots.

### 2.3 Cell-cell communication network analysis

Cell-cell communication network analysis was performed using the CellChat R package to compare HC vs UC and HC vs CD separately (38). Cells were grouped by major cell types from Seurat object, and CellChat objects were constructed from the log-normalized expression matrix (Seurat RNA assay @data) using CellChatDB.human. Overexpressed genes and ligand–receptor interactions were identified using identifyOverExpressedGenes and identifyOverExpressedInteractions. Communication probabilities were inferred using computeCommunProb (raw.use = TRUE; population.size = TRUE), followed by pathway-level computation (computeCommunProbPathway) and network aggregation (aggregateNet). Two conditions were merged using mergeCellChat, and differences in interaction number and strength were quantified using compareInteractions and visualized with netVisual_diffInteraction and netVisual_heatmap.

### 2.4 Gene set enrichment analysis

To further explore cellular functional changes during IBD disease progression,GSEA was performed using the “ClusterProfiler” R package on each major cell population separately (39, 40). By comparing transcriptomic differences between HC and CD, as well as between HC and UC, we aimed to identify which specific cell populations underwent significant biological functional alterations during the pathological progression of UC and CD. In the analytical workflow, the “FindMarkers” function was first employed to identify differentially expressed genes within each cell population. Genes were then ranked by log2FC values to construct ordered gene lists, followed by enrichment analysis based on the “MSigDB Hallmark gene sets” (41). A significance threshold of adjusted p-value < 0.05 was applied, and enrichment results were visualized using the dotplot function.

### 2.5 Myeloid subpopulation analysis

To further characterize myeloid cell heterogeneity, motivated by the enrichment of shared DEGs in immune compartments, myeloid cells were subset from the integrated dataset and re-analyzed. Dimensionality reduction was performed by PCA (20 principal components), followed by graph-based re-clustering (resolution = 0.2). Marker genes for each subcluster were identified using Seurat FindAllMarkers (min.pct = 0.25; logfc.threshold = 0.25). Myeloid subclusters were annotated into six transcriptionally defined states based on canonical markers: Classical_Mye (*TMSB4X, AIF1, TYROBP*), Inflammatory_Mye (*IL1B, VCAN, FCN1*), Complement_Mye (*APOE, CTSC, C1QB*), Transcription_Mye (*FOS, JUN, RGS1*), Chemokine_Mye (*CXCL8, IFITM2, NAMPT*), and Cytotoxic_Mye (*CST7, RUNX3, TCTN3*). Differences in the relative abundance of myeloid subpopulations across HC, UC, and CD were assessed. In IBD-enriched myeloid subsets, we further evaluated the co-expression relationships between *IL1B* and chemokine genes, constructed chemokine-centered PPI networks to prioritize candidate regulators, and examined their expression patterns across myeloid subpopulations.

### 2.6 Animal experimental validation

#### 2.6.1 Construction of rat colitis and periodontitis models

Healthy adult Sprague–Dawley (SD) rats were randomly divided into four groups (n = 3 per group): a control group without treatment; a periodontitis group in which periodontitis was induced by ligating the buccal gingiva of the maxillary first molar with silk thread for 14 consecutive days (42); an IBD group in which ulcerative colitis was induced by administering 3% dextran sulfate sodium (DSS) in drinking water for 7 consecutive days followed by normal water (43); and a periodontitis + IBD group in which both conditions were induced simultaneously using the above methods. At the end of the experimental period, serum and mid-colon tissues were collected for subsequent analyses. All animal procedures were approved by the Animal Ethics Committee of Binzhou Medical University (Approval No.: 2025-L024) and were conducted in accordance with relevant institutional guidelines and regulations.

#### 2.6.2 ELISA

Serum concentrations of rat interleukin-1β (IL1β) and C-X-C motif chemokine ligand 2 (CXCL2) were measured using commercial ELISA kits (IL1B: JL20884-96T, Jianglai Biotechnology; CXCL2: AB-K260303, Abmart) according to the manufacturers’ protocols. Standard, blank, and sample wells were prepared. Standards (50 μL) of varying concentrations were added to the standard wells, while no sample or enzyme-labeled reagent was added to the blank wells. Serum samples were diluted 1:5 with sample diluent, and 50 μL of the diluted samples were added to sample wells, avoiding contact with the well walls, and gently mixed. Enzyme-labeled reagent (100 μL) was added to each well except blanks, and the plates were sealed and incubated at 37°C for 60 min. After incubation, plates were washed five times with a 1× washing buffer prepared from a 20× stock solution, allowing 30 s soaking each time. Subsequently, 50 μL of chromogen A and 50 μL of chromogen B were sequentially added to each well, gently mixed, and incubated at 37°C in the dark for 15 min. The reaction was terminated by adding 50 μL of stop solution, and absorbance was measured at 450 nm within 15 min using a microplate reader. Optical density (OD) values were converted to concentrations using a standard curve generated from serial dilutions of the standards. Curve fitting was performed using a four-parameter logistic (4PL) regression model, and concentrations of samples were interpolated from the fitted curve. All measurements were performed in three times, and the mean values were used for statistical analysis.

#### 2.6.3 Immunohistochemistry

Colon tissues were fixed, embedded in paraffin, and sectioned at 5 μm thickness. Sections were deparaffinized in xylene, rehydrated through graded ethanol, and treated with 3% H_2_O_2_ in methanol to block endogenous peroxidase activity. Antigen retrieval was performed in citrate buffer at 100°C for 10 min, followed by cooling to room temperature and blocking with 5% bovine serum albumin for 20 min. Primary antibodies against IL1β (16806-1-AP, Proteintech) and CXCL2 (83758-1-RR, Proteintech) were applied and incubated overnight at 4°C. After three washes in PBS (5 min each), horseradish peroxidase–conjugated goat anti-rabbit IgG (33101ES60, YEASEN) diluted in PBS was applied for secondary incubation at room temperature. Detection was carried out using DAB substrate (P0203, Beyotime) for 3 min, followed by hematoxylin counterstaining (C0107, Beyotime) for 3 min and differentiation in acid alcohol for 1 min. Slides were dehydrated through graded ethanol, cleared in xylene, and mounted with neutral resin. Images were acquired under a light microscope at 40× to 200× magnification. Immunohistochemical staining intensity was evaluated using a four-tier scoring system: 0 (negative, no positive staining), 1 (weak positive, light yellow), 2 (positive, brown-yellow), and 3 (strong positive, dark brown) (27). The H-score was calculated using the formula: H-score = 0×(percentage of negative cells) + 1×(percentage of weakly positive cells) + 2×(percentage of moderately positive cells) + 3×(percentage of strongly positive cells), with scores ranging from 0-300. For area-based quantification, digital image analysis was performed to measure the positively stained area and total area within regions of interest (ROI). The percentage of positively stained area (%Area = positive area/total area × 100%) was calculated as the primary quantitative metric for statistical analysis (44).

## 3 Results

### 3.1 Transcriptomic profiling of periodontal tissues

**Figure 1:**
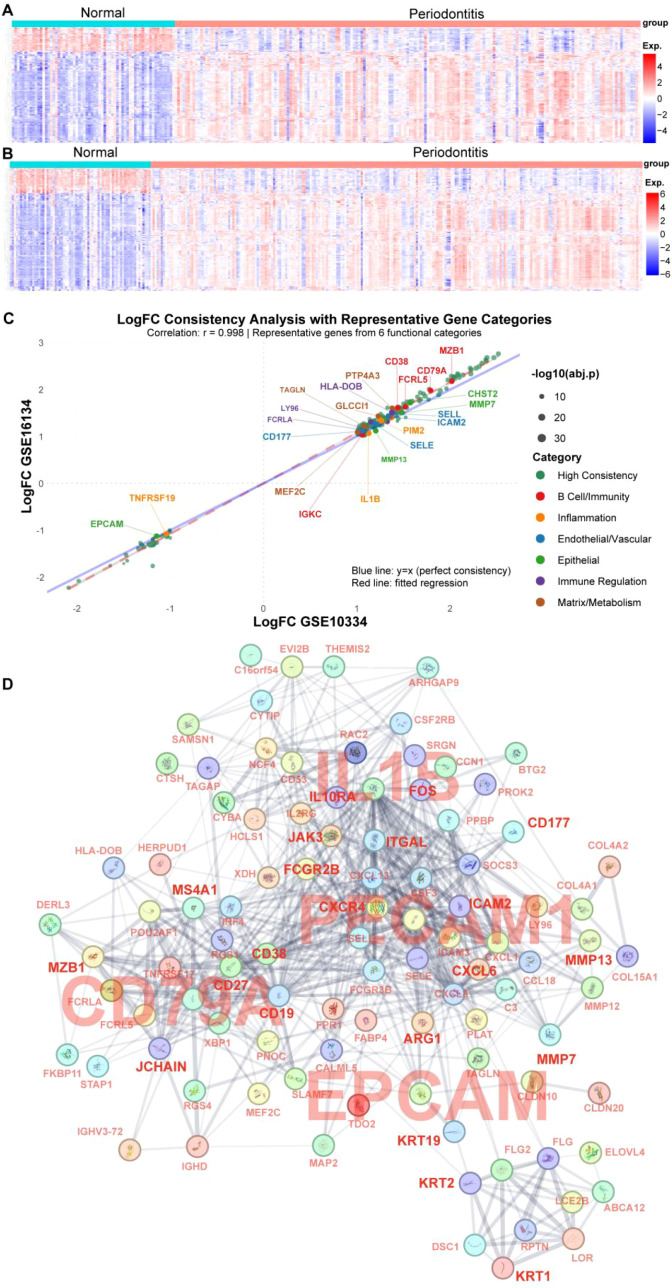
Differential gene expression analysis and protein-protein interaction network comparing periodontitis lesions with normal tissues. **(A, B)** Heatmaps of differentially expressed genes from datasets GSE10334 **(A)** and GSE16134 **(B)**, where blue represents low expression and red represents high expression. **(C)** Consistency analysis of upregulated and downregulated genes commonly found in datasets GSE10334 and GSE16134 in lesional tissues, along with functional classification of signature genes. **(D)** Protein-protein interaction (PPI) network of commonly differentially expressed genes from datasets GSE10334 and GSE16134. Key signature genes are highlighted and magnified to emphasize critical regulatory networks of different cell types: CD79A represents B cell lineage, IL1B represents inflammatory regulation pathways, PECAM1 represents endothelial cells, and EPCAM represents epithelial cells. Other important signature genes associated with various cell populations are similarly emphasized in red bold font.

**Figure 2:**
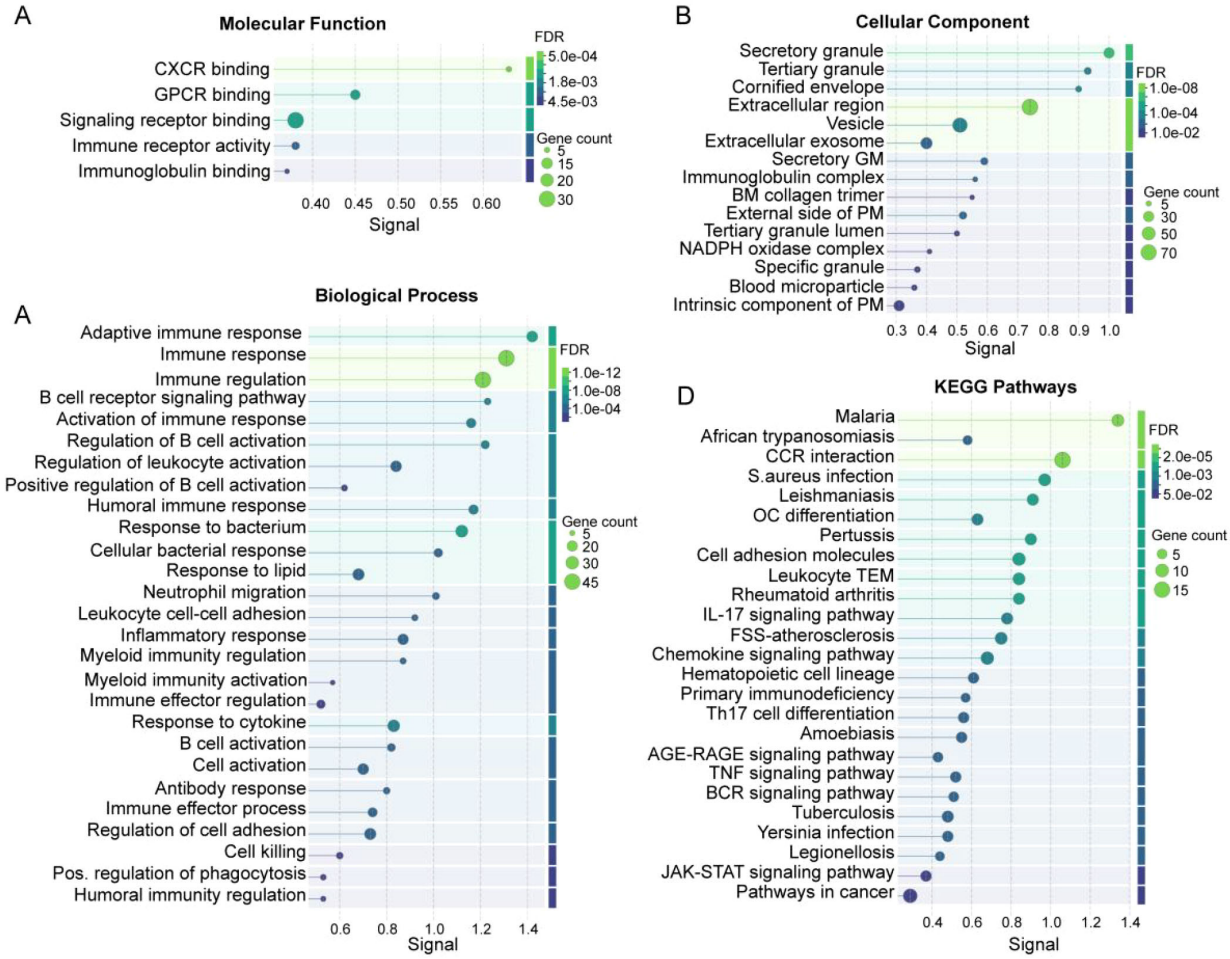
Functional enrichment analysis results of protein-protein interaction (PPI) networks constructed from commonly differentially expressed genes (DEGs) in datasets GSE10334 and GSE16134 based on the STRING database. **(A-C)** Gene Ontology (GO) analysis of commonly DEGs, including molecular function **(A)**, cellular component **(B)**, and biological process **(C)**. **(D)** KEGG pathway enrichment analysis of commonly DEGs. FDR, false discovery rate; CXCR, chemokine receptor binding; GPCR, G protein-coupled receptor binding; GM, granule membrane; BM, basement membrane; PM, plasma membrane; CCR, cytokine-cytokine receptor; OC, osteoclast differentiation; TEM, transendothelial migration; FSS, fluid shear stress and atherosclerosis; BCR, B cell receptor.

In GSE16134, 249 DEGs were identified between periodontitis and healthy tissues (198 upregulated, 51 downregulated; **Figure 1A**, **Supplementary Table S1**), while GSE10334 yielded 190 DEGs (150 upregulated, 40 downregulated; **Figure 1B**, **Supplementary Table S2**). Intersecting the two cohorts identified 188 common DEGs (153 upregulated, 35 downregulated; **Supplementary Table S3**), with concordant directions of change across datasets (**Figure 1C**), supporting the robustness of the periodontitis signature. The 188 DEGs mapped to multiple immune and tissue modules rather than a single pathway. B cell–related genes (*CD38, CD79A, MZB1*) were upregulated, consistent with enhanced humoral immune activation. Inflammation-related genes including *IL1B* were among the most prominent upregulated signals, supporting an inflammatory microenvironment. Endothelial adhesion/trafficking markers (*CD177, SELE, ICAM2, SELL*) were increased, consistent with leukocyte recruitment and vascular activation. In contrast, *EPCAM* was reduced, suggesting epithelial barrier disruption, whereas remodeling-associated genes (*MMP7, MMP13, CHST2*) were elevated, indicating extracellular matrix remodeling. PPI network analysis further resolved these DEGs into distinct clusters (**Figure 1D**), including immune activation, inflammatory cytokine/chemokine signaling, endothelial activation, and epithelial/tissue remodeling modules. Functional enrichment analysis (**Figure 2**) showed that in the MF category, periodontitis-related DEGs were enriched in chemokine receptor–related binding activities (including CXCR binding and GPCR binding), immune receptor activity, and immunoglobulin binding (**Figure 2A**). BP analysis highlighted immune processes spanning adaptive immunity (e.g., B cell receptor signaling) and innate immunity (e.g., leukocyte activation, neutrophil migration, and regulation of myeloid immune responses). CC analysis indicated enrichment in secretory granules and extracellular regions, consistent with a secreted inflammatory milieu. KEGG pathway analysis identified inflammation- and immunity-related pathways, including chemokine signaling, IL-17 signaling, TNF signaling, and B cell receptor signaling, alongside infection-associated signatures. Together, these data delineate a robust periodontal inflammatory transcriptomic program characterized by chemokine/cytokine signaling and coordinated immune–stromal alterations, which provides a molecular basis for cross-disease comparisons with intestinal inflammation.

### 3.2 Overview of single-cell transcriptomic data in IBD

**Figure 3:**
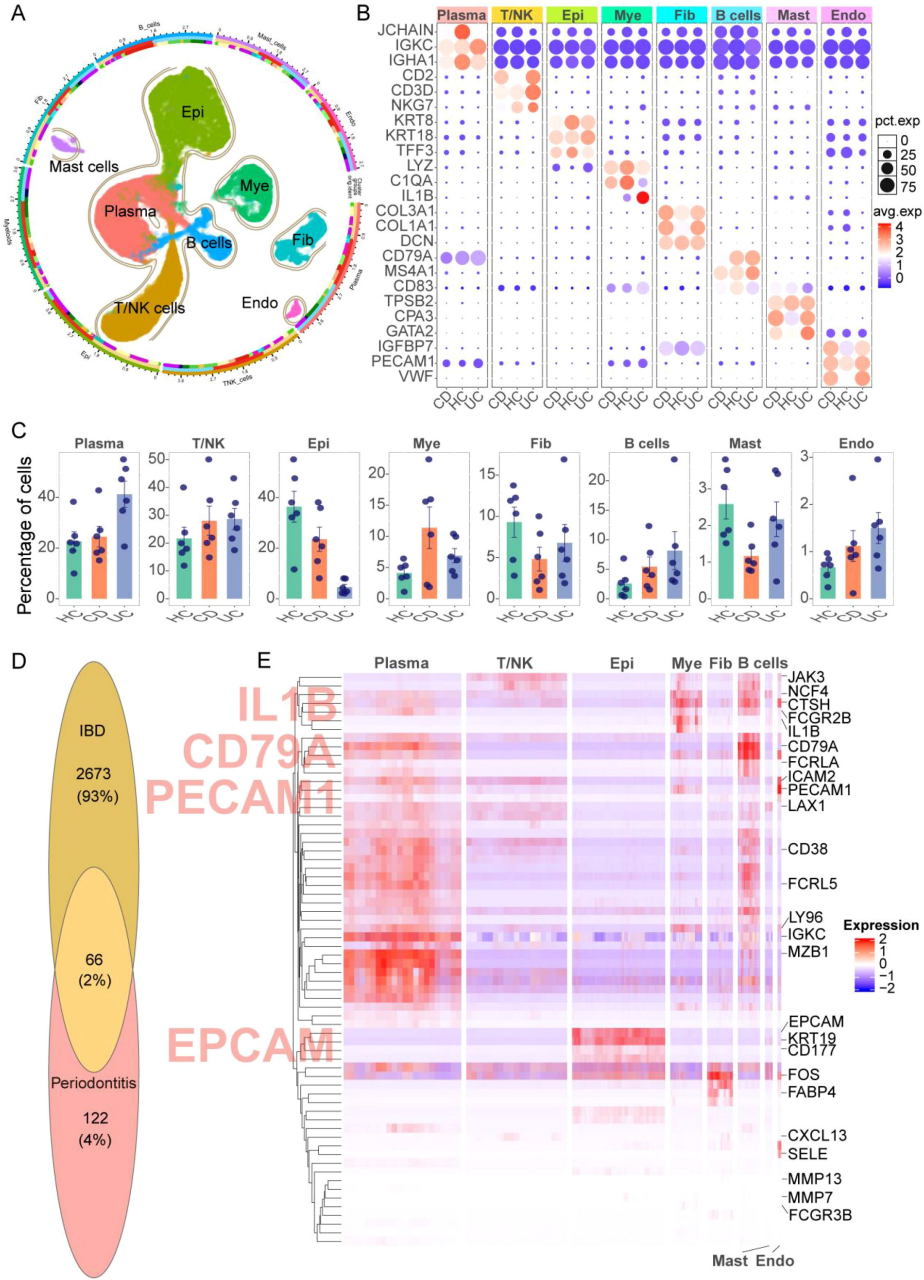
Single-cell transcriptomic landscape of IBD and cell-type distribution of genes shared with periodontitis. **(A)** UMAP visualization of the IBD single-cell atlas (GSE214695), including healthy controls (HC), Crohn’s disease (CD), and ulcerative colitis (UC) samples. **(B)** Dot/heatmap showing canonical marker gene expression used to annotate major cell populations. **(C)** Proportional changes of major cell populations across HC, CD, and UC, with each dot representing one sample. **(D)** Venn diagram showing the overlap between IBD-associated differentially expressed genes identified by pseudobulk analysis (DESeq2; aggregated by sample and condition) and periodontitis DEGs derived from bulk microarray meta-comparison, yielding 66 shared DEGs (**Supplementary Table S4**). **(E)** Heatmap showing the cell-type–resolved expression pattern of the 66 shared DEGs across the major cell populations in GSE214695 (scaled expression), where red indicates higher and blue indicates lower expression. The shared genes are detected across multiple cell types but show prominent expression in immune compartments (including myeloid and plasma/B-lineage cells), supporting a data-driven focus on myeloid programs in downstream analyses. Epi, epithelial cells; Mye, myeloid cells; Fib, fibroblasts; Endo, endothelial cells; T/NK, T cells/natural killer cells; HC, healthy controls; CD, Crohn’s disease; UC, ulcerative colitis.

The IBD single-cell RNA-seq dataset contained 51,322 cells, comprising 16,949 from healthy controls (HC), 19,558 from CD, and 14,815 from UC. Cells were clustered into eight major populations (**Figure 3A**), each defined by characteristic marker genes with consistent expression patterns across disease states (**Figure 3B**). In terms of abundance (**Figure 3C**), plasma cells were most frequent, followed by T/NK cells, epithelial cells, myeloid cells, fibroblasts, B cells, mast cells, and endothelial cells. Relative to HC, UC samples exhibited an increased proportion of plasma cells, whereas both CD and UC showed higher fractions of T/NK and myeloid cells, consistent with immune-enriched inflammatory microenvironments. To identify IBD-associated transcriptional changes while reducing cell-level pseudo-replication, we performed sample-level pseudobulk differential expression analysis, which yielded 2,739 IBD-related DEGs. Intersecting these DEGs with the 188 consensus periodontitis DEGs identified 66 shared genes (**Figure 3D**, **Supplementary Table S4**). We then mapped these 66 shared genes back to the single-cell atlas to determine their cell-type distribution. Although the 66 shared genes were detectable across all eight cell types, they segregated into distinct cell-type–biased modules, with a prominent plasma/B-cell program (e.g., *IGKC, MZB1, CD38, FCRL5, CD79A*) and an innate inflammatory myeloid program centered on *IL1B* (e.g., *IL1B, NCF4, CTSH, FCGR2B/FCGR3B, LAX1*), alongside epithelial (*EPCAM, KRT19*) and endothelial (*PECAM1, ICAM2, SELE*) signatures (**Figure 3E**). This modular distribution provided a data-driven rationale for prioritizing myeloid cells for downstream analyses, given the clear IL1B-centered inflammatory signal in the myeloid compartment.

### 3.3 Cell-cell communication analysis

#### 3.3.1 Overview of cell-cell communication

**Figure 4:**
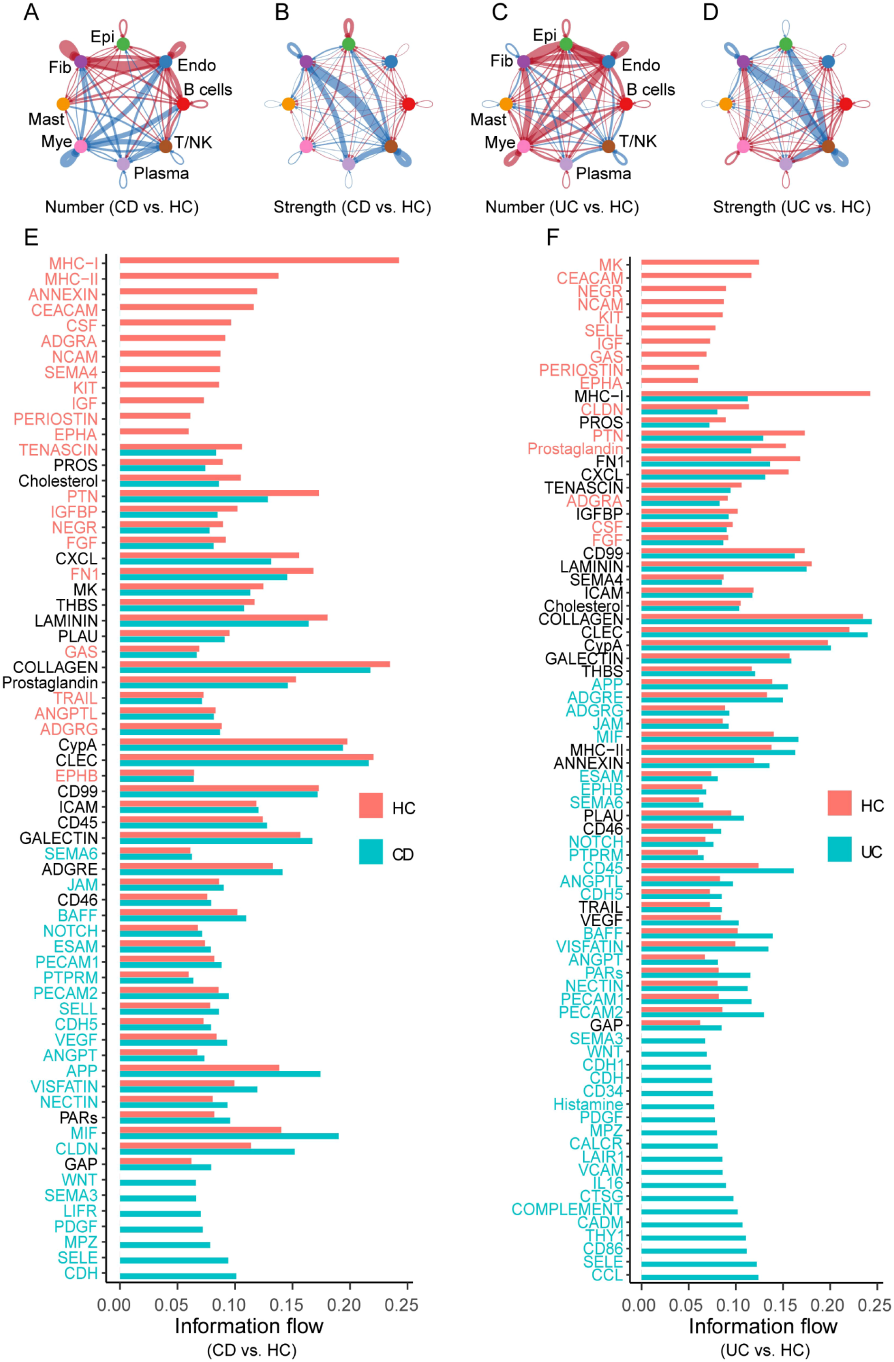
Cell–cell communication remodeling in IBD highlights myeloid-centered signaling and inflammatory/ECM pathways. **(A–D)** Circle plots summarizing the number **(A, C)** and overall interaction strength **(B, D)** of inferred intercellular communications across major cell types in CD vs. HC **(A, B)** and UC vs. HC **(C, D)** comparisons. Line thickness denotes interaction strength; node colors indicate cell populations. **(E, F)** Bar plots showing information flow (overall pathway-level communication strength) for signaling pathways in CD vs. HC **(E)** and UC vs. HC **(F)** comparisons, with red bars representing HC and blue bars representing CD or UC. Across both IBD subtypes, immune-related pathways (e.g., MHC-I/MHC-II, ANNEXIN) and extracellular matrix–associated programs (e.g., COLLAGEN, TENASCIN) exhibited increased information flow, consistent with immune activation and tissue remodeling. Notably, chemokine-related signaling (e.g., CXCL pathway) also showed elevated activity, aligning with the shared inflammatory module identified in the periodontitis bulk transcriptomes and supporting a testable myeloid–chemokine axis relevant to oral–gut inflammatory crosstalk. Epi, epithelial cells; Mye, myeloid cells; Fib, fibroblasts; Endo, endothelial cells; T/NK, T cells/natural killer cells.

To characterize disease-associated remodeling of intercellular signaling in IBD, CellChat was applied to compare HC with CD and UC. Both disease groups exhibited increased network connectivity and interaction strength relative to HC (**Figure 4**). Myeloid cells consistently emerged as prominent communication hubs, together with plasma and epithelial compartments. Pathway-level analysis revealed that disease states were characterized by coordinated immune activation and stromal remodeling signals, including antigen presentation–related pathways (*MHC-I/MHC-II*), chemokine signaling (e.g., *CXCL/CCL*), and extracellular matrix–associated programs (e.g., *COLLAGEN/FN1/TENASCIN*) (**Figures 4E, F**). Notably, the enrichment of chemokine-related communication aligns with the chemokine/CXCR-binding signature observed in periodontitis bulk transcriptomes, supporting a shared inflammatory axis across the oral–gut context.

#### 3.3.2 Incoming signaling patterns

**Figure 5:**
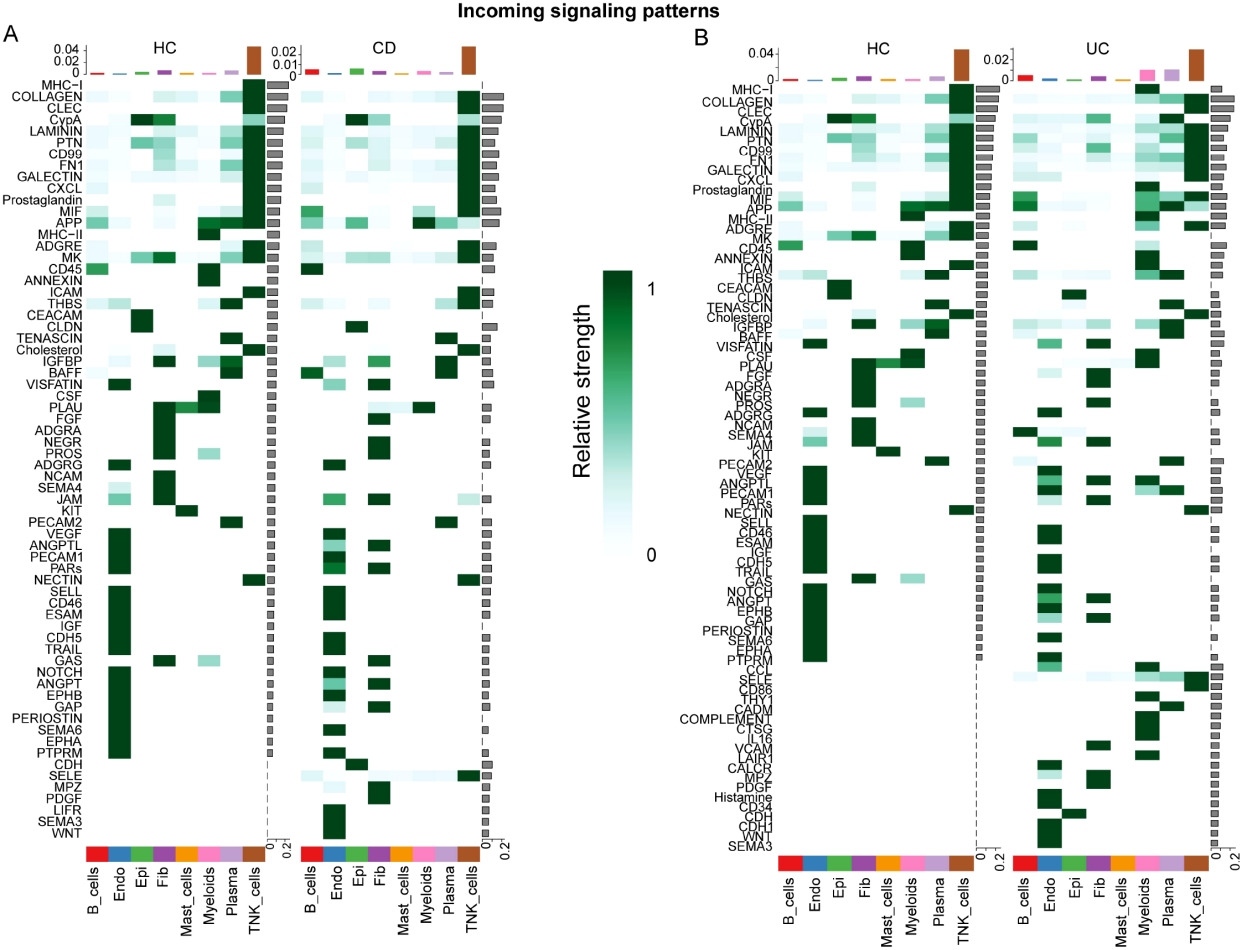
Incoming signaling patterns highlight disease-associated shifts in signal reception across cell types in IBD. **(A, B)** Heatmaps showing incoming (receiver) signaling roles inferred by CellChat in CD vs. HC **(A)** and UC vs. HC **(B)** comparisons. Rows denote signaling pathways and columns denote cell types; color intensity reflects the relative strength of received signals (information flow), with darker colors indicating stronger reception. Across both IBD subtypes, myeloid cells exhibited prominently increased signal reception, with additional increases observed in plasma and epithelial compartments, consistent with a myeloid-centered communication architecture in inflamed tissues. Pathway-level differences between CD and UC indicate subtype-specific rewiring, including immune-related (e.g., *MHC*) and chemokine-associated routes (e.g., *CXCL*), which align with the inflammatory module shared with periodontitis. Epi, epithelial cells; Mye, myeloid cells; Fib, fibroblasts; Endo, endothelial cells; T/NK, T cells/natural killer cells.

Incoming signaling analysis showed a disease-associated increase in signal reception across multiple immune compartments (**Figures 5A, B**). In CD, myeloid, plasma, and epithelial cells displayed enhanced reception, particularly in antigen presentation and matrix-related pathways. In UC, increased reception was also evident in myeloid and plasma cells, with additional contributions from lymphoid compartments (B cells and T/NK cells) through inflammatory programs such as *CCL*/*COMPLEMENT*/*IL16*, and increased endothelial involvement, consistent with vascular activation in UC.

#### 3.3.3 Outgoing signaling patterns

**Figure 6:**
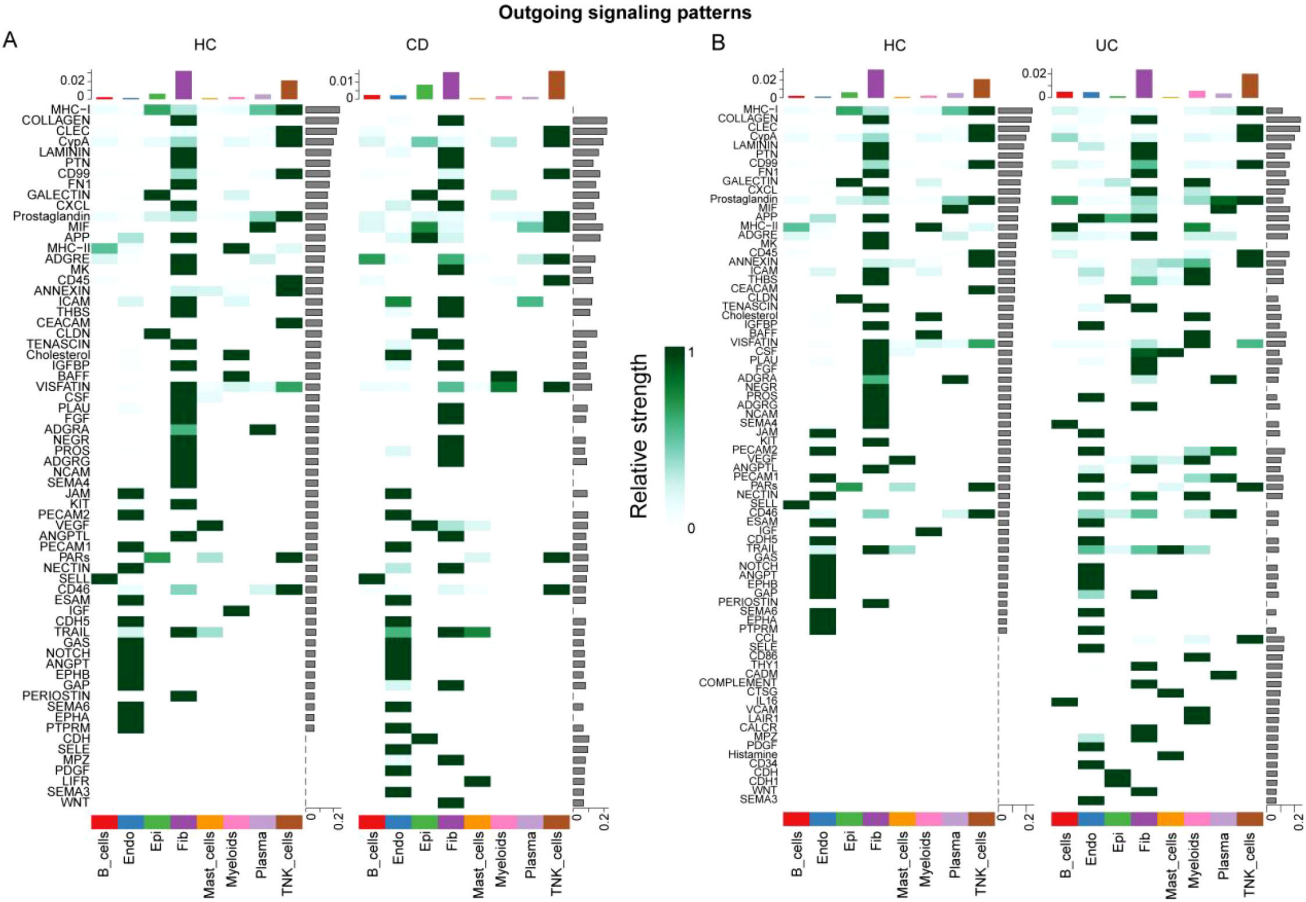
Outgoing signaling patterns indicate myeloid-dominant signal sending in IBD. **(A, B)** Heatmaps showing outgoing (sender) signaling roles inferred by CellChat in CD vs. HC **(A)** and UC vs. HC **(B)** comparisons. Rows denote signaling pathways and columns denote cell types; color intensity reflects the relative strength of transmitted signals (information flow), with darker colors indicating stronger signal output. Across both IBD subtypes, myeloid cells displayed the strongest increase in outgoing signaling, supporting their role as dominant coordinators of intercellular communication in inflamed tissues. Plasma cells and endothelial cells exhibited pathway-specific increases in signal output, consistent with immune activation and vascular/tissue remodeling features in IBD. Enhanced output in immune-related and chemokine-associated pathways (e.g., MHC and CXCL) provides a communication-level correlate of the shared inflammatory signatures identified across periodontitis and IBD. Epi, epithelial cells; Mye, myeloid cells; Fib, fibroblasts; Endo, endothelial cells; T/NK, T cells/natural killer cells.

Outgoing signaling analysis indicated that myeloid cells were major signal senders in both CD and UC (**Figures 6A, B**). In CD, myeloid output was dominated by antigen presentation–associated pathways and chemokine/ECM-related signaling, whereas in UC, myeloid cells showed stronger output in inflammatory pathways (e.g., *CCL*/*COMPLEMENT*/*IL16*). Plasma cells displayed increased output in B-cell–associated regulatory programs, while endothelial cells in UC exhibited enhanced vascular-related signaling (e.g., *ANGPT*/*VEGF*), consistent with heightened endothelial activation. Together, these communication patterns highlight a myeloid-centered inflammatory signaling network with prominent chemokine components, providing a mechanistic link to the shared IL1B–chemokine signature identified across periodontitis and IBD.

### 3.4 GSEA

**Figure 7:**
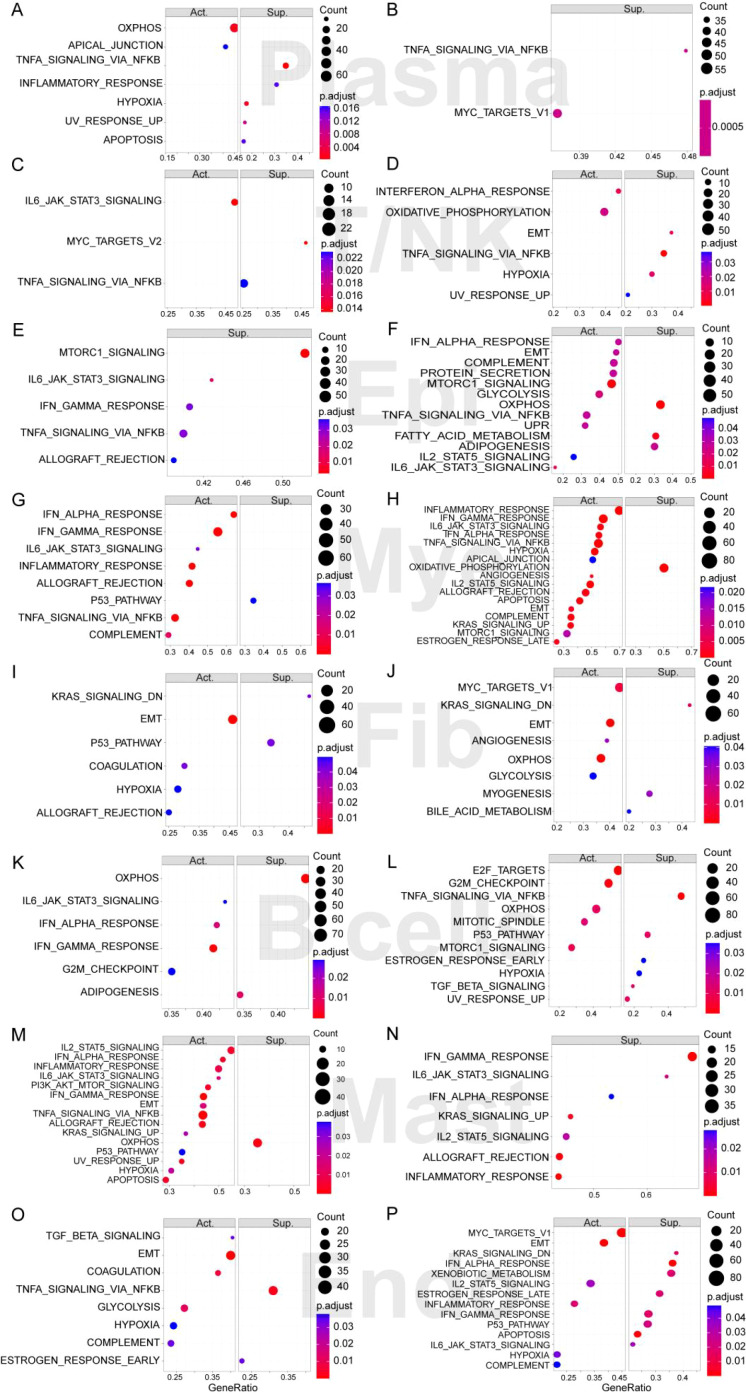
GSEA reveals myeloid cells as central mediators of shared pathological pathways. **(A-P)** Bubble plots showing GSEA results for different cell types: **(A)** Plasma cells, **(B)** T/NK cells, **(C)** Epithelial cells, **(D)** Myeloid cells, **(E)** Fibroblasts, **(F)** B cells, **(G)** Mast cells, and **(H)** Endothelial cells. Each bubble represents a significantly enriched pathway, with bubble size indicating gene count and color intensity representing adjusted p-value. Myeloid cells **(H)** show the most extensive and significant pathway enrichment, particularly in inflammatory response (TNFA_SIGNALING_VIA_NFKB, INFLAMMATORY_RESPONSE), immune activation (IL6_JAK_STAT3_SIGNALING, IFN_GAMMA_RESPONSE), and complement pathways, highlighting their central role in IBD pathogenesis.

GSEA of shared DEGs across cell types (**Figures 7A–P**) revealed that myeloid cells had the most extensive and significant pathway enrichment (**Figures 7G, H**), including classical inflammatory (TNFA_SIGNALING_VIA_NFKB, INFLAMMATORY_RESPONSE, IL6_JAK_STAT3_SIGNALING), adaptive immune (ALLOGRAFT_REJECTION, IFN_GAMMA_RESPONSE, IFN_ALPHA_RESPONSE), and complement (COMPLEMENT) pathways. Metabolic reprogramming signatures (OXIDATIVE_PHOSPHORYLATION, MTORC1_SIGNALING) and stress-response pathways (HYPOXIA, APOPTOSIS) were also prominent, reflecting high energy demand and adaptation to inflammatory stress. Other cell types showed more restricted enrichments: plasma cells in immunoglobulin-related pathways, T/NK cells in cytotoxic and immune activation pathways, and epithelial cells in junction/barrier function pathways. Collectively, these results identify myeloid cells as central hubs in both sending and receiving signals, with broad functional activation positioning them as a key molecular bridge linking periodontitis and IBD.

### 3.5 Subpopulation characteristics of myeloid cells

**Figure 8:**
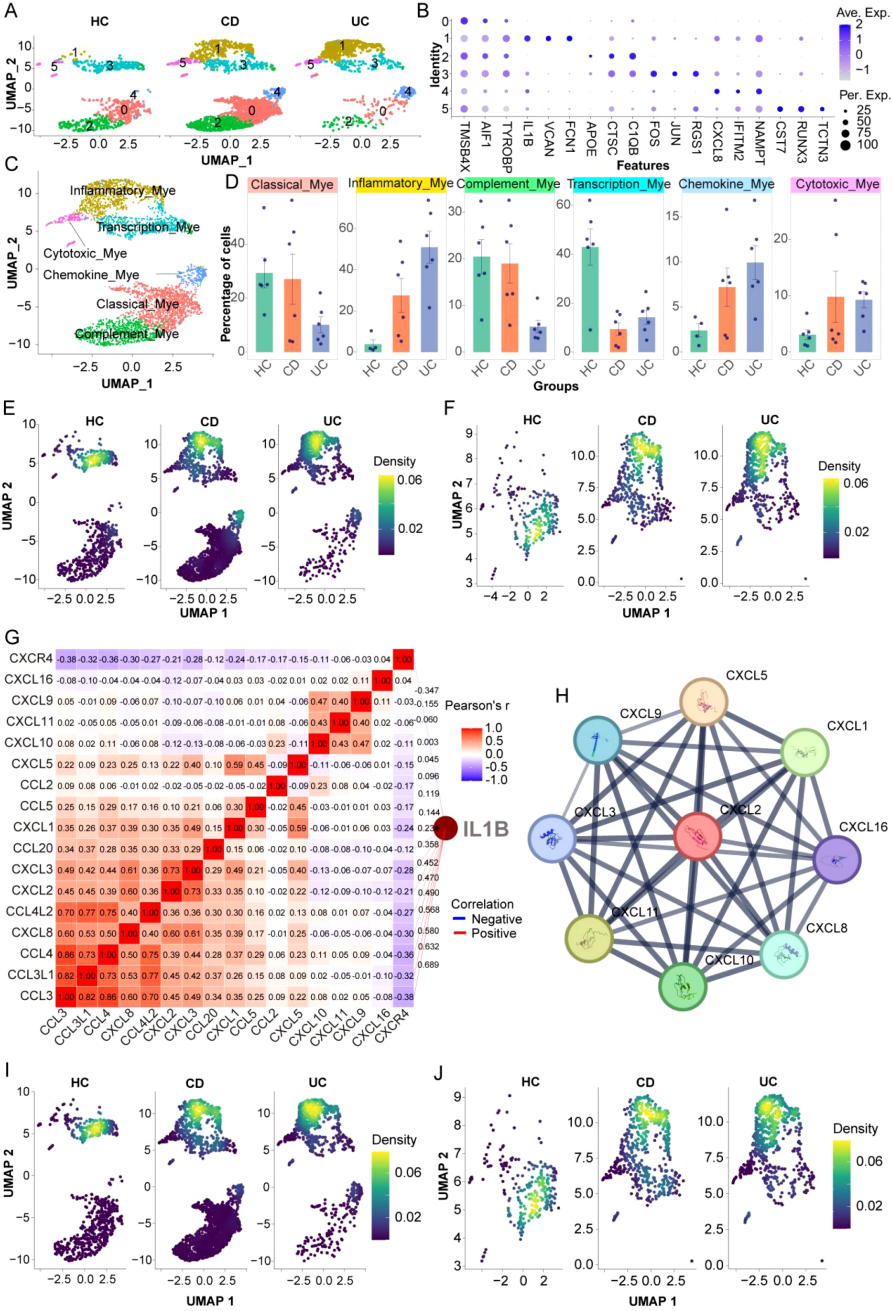
Heterogeneity and functional specialization analysis of myeloid cell (Mye) subpopulations in inflammatory bowel disease. **(A)** Six subpopulations identified through myeloid cell subcluster analysis and their distribution in healthy controls (HC), Crohn’s disease (CD), and ulcerative colitis (UC). **(B)** Heatmap of marker gene expression for each subpopulation, where blue represents high expression. **(C)** Classification of myeloid cells based on marker gene expression into Classical_Mye, Inflammatory_Mye, Complement_Mye, Transcription_Mye, Chemokine_Mye, and Cytotoxic_Mye. **(D)** Proportion analysis of myeloid cell subpopulations in HC, CD, and UC, with each dot representing an individual sample. **(E, F)** Density distribution characteristics of inflammatory **(E)** and chemokine-related **(F)** myeloid cells in disease states. **(G)** Chemokine correlation matrix analysis centered on IL1B, where red indicates positive correlation, blue indicates negative correlation, and numerical values represent correlation coefficient r values. **(H)** Protein-protein interaction (PPI) analysis of chemokine-mediated inflammatory networks. **(I, J)** UMAP-based spatial mapping analysis showing the expression distribution of neutrophil chemokine CXCL2 across myeloid cell subpopulations in HC, CD, and UC **(I)**, and highlighting the high CXCL2 expression in transcription-regulatory myeloid cell subpopulations in HC group and the spatially clustered high expression pattern of CXCL2 in inflammatory myeloid cell subpopulations in CD and UC groups **(J)**.

To further resolve myeloid heterogeneity in IBD, we re-clustered myeloid cells and identified six transcriptionally distinct subpopulations (**Figures 8A–C**): Classical_Mye, Inflammatory_Mye, Complement_Mye, Transcription_Mye, Chemokine_Mye, and Cytotoxic_Mye. Proportion analysis revealed disease-associated compositional shifts (**Figure 8D**). Inflammatory_Mye increased in both CD and UC, whereas Complement_Mye expanded more prominently in UC. Chemokine_Mye was elevated in both subtypes and appeared more pronounced in CD, consistent with enhanced inflammatory signaling in disease tissues. Density mapping further showed that Inflammatory_Mye and Chemokine_Mye displayed clustered distributions in CD/UC compared with HC (**Figures 8E, F**), supporting spatially coordinated inflammatory niches. To prioritize candidate mediators within myeloid inflammation, we examined chemokine–inflammatory gene relationships. Correlation analysis identified IL1B as a central node positively associated with multiple chemokine genes (e.g., *CCL3, CCL4, CXCL8*) (**Figure 8G**), and PPI analysis confirmed these molecules form a tightly connected chemotactic network (**Figure 8H**). *CXCL2* was selected for downstream validation because its expression showed clear myeloid-state specificity and disease-associated upregulation: UMAP mapping demonstrated that *CXCL2*-high cells localized predominantly to Inflammatory_Mye and Transcription_Mye, with limited expression in other subpopulations (**Figure 8I**). While *CXCL2* expression in HC was mainly confined to Transcription_Mye, CD and UC showed a marked increase of *CXCL2* within Inflammatory_Mye, accompanied by an evident clustered distribution pattern (**Figure 8J**). Together, these results support an *IL1B*-centered inflammatory chemokine program within disease-enriched myeloid states, highlighting *CXCL2* as a representative effector chemokine for *in vivo* consistency assessment.

### 3.6 Expression of IL1B and CXCL2 in the rat disease models

**Figure 9:**
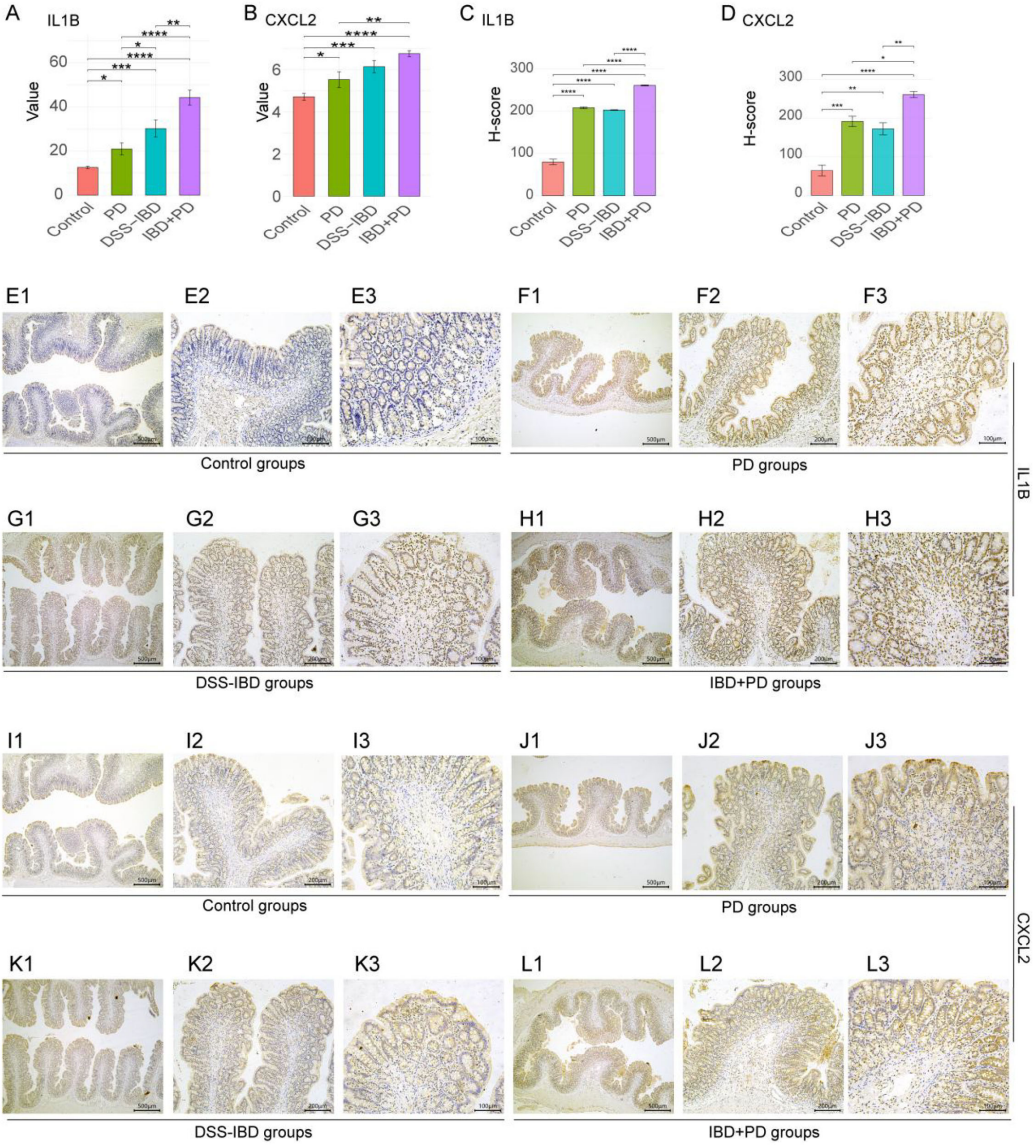
Expression validation of IL1B and CXCL2 in rat disease models. **(A)** Serum IL-1β concentration measurements across experimental groups. **(B)** Serum CXCL2 concentration measurements across experimental groups. **(C)** Quantitative analysis of IL1B immunohistochemical staining in colonic tissues (%Area). **(D)** Quantitative analysis of CXCL2 immunohistochemical staining in colonic tissues (%Area). **(E-H)** Representative micrographs of IL1B expression in colonic tissues across experimental groups (multiple magnifications). **(I-L)** Representative micrographs of CXCL2 expression in colonic tissues across experimental groups (multiple magnifications). Numbers 1, 2, and 3 represent resolutions of 500μm, 200μm, and 100μm, respectively. Experimental groups: Control, periodontitis (PD), dextran sulfate sodium-induced inflammatory bowel disease (DSS-IBD), and inflammatory bowel disease combined with periodontitis (IBD+PD). Statistical analysis performed using one-way ANOVA with Tukey *post hoc* test, adjusted p < 0.05 indicates statistical significance. *, p<0.05; **, p<0.01; ***, p<0.001; ****, p<0.0001.

To evaluate whether the *IL1B–CXCL2* axis observed in human transcriptomic analyses could also be detected *in vivo* under combined oral and intestinal inflammation, we quantified IL-1β and CXCL2 in the rat models. Serum IL-1β levels were significantly higher in the PD, DSS-IBD, and IBD+PD groups than in Controls, with the greatest increase observed in IBD+PD (**Figure 9A**; one-way ANOVA with Tukey *post hoc*, adjusted p < 0.05). CXCL2 showed a concordant pattern, with all disease groups exceeding Controls and the IBD+PD group displaying the highest concentrations (**Figure 9B**; adjusted p < 0.05).

Quantitative immunohistochemistry demonstrated increased IL-1β (**Figures 9C, E–H**) and CXCL2 (**Figures 9D, I–L**) staining in PD and DSS-IBD, with the most pronounced increases in IBD+PD (**Figures 9C, D**; ANOVA with Tukey, adjusted p < 0.05). These data provide *in vivo* consistency evidence that the *IL1B–CXCL2* inflammatory axis is elevated when periodontal and intestinal inflammation coexist. Given the small sample size and the combined-stimulus design, these findings should be interpreted as validation of concordant inflammatory activation rather than mechanistic proof of therapeutic causality. Quantitative metrics, including %Area and H-scores, are provided in **Supplementary Table S5**.

## 4 Discussion

This study integrated bulk gingival transcriptomes and single-cell RNA sequencing to delineate molecular programs shared between periodontitis and IBD and to map their cell type–biased expression patterns. By integrating two independent periodontitis cohorts, we identified 188 common DEGs with concordant changes in diseased tissues, providing a robust foundation for downstream pathway interrogation. Across functional module and PPI network structure, B-cell–related genes (e.g., *CD38, CD79A, MZB1*) were consistently upregulated, whereas epithelial markers (e.g., *EPCAM*) were downregulated. In parallel, inflammatory mediators including *IL1B* and *PIM2* were increased, and endothelial adhesion molecules (*CD177, SELE, ICAM2, SELL*) were elevated, collectively indicating coordinated immune activation with epithelial barrier alteration and vascular activation in periodontitis. To examine potential molecular overlap with intestinal inflammation, pseudobulk differential analysis of IBD scRNA-seq data (51,322 cells) identified 2,739 disease-associated DEGs, and intersecting these with periodontitis DEGs yielded 66 shared genes. Mapping these shared genes back to the single-cell atlas revealed a modular, cell-type–biased pattern, with prominent signals in plasma cells/B cells, epithelial cells, and a distinct innate inflammatory module in myeloid cells. Consistently, CellChat analysis highlighted myeloid cells as major hubs within remodeled IBD communication networks, and GSEA supported enrichment of inflammatory programs (TNFA_SIGNALING_VIA_NFKB, INFLAMMATORY_RESPONSE, IL6_JAK_STAT3_SIGNALING) in myeloid compartments. Myeloid cells further resolved into six transcriptionally defined subpopulations, with inflammatory- and chemokine-associated states expanded in CD and UC and complement-associated cells relatively more prominent in UC. Within disease-enriched myeloid states, *IL1B* emerged as a central node linked to multiple chemokine genes (including *CXCL2, CXCL8, CCL3*, and *CCL4*), suggesting a coordinated IL1B–chemokine inflammatory program rather than isolated single-gene effects. Importantly, our *in vivo* experiments were designed to assess consistency with the transcriptomic signatures rather than to establish mechanistic causality. In rat models, serum ELISA and colon immunohistochemistry showed increased IL-1β protein and CXCL2 expression, with the highest levels observed in the combined IBD+PD group. Together, these results support the presence of a shared, myeloid-centered inflammatory network across oral and intestinal inflammation, providing molecular support for oral–gut axis crosstalk. Given the cross-sectional nature of the public datasets and the small-sample, combined-stimulus animal design, functional perturbation studies will be required to determine whether targeting the IL-1β–chemokine axis yields therapeutic benefit.

From a clinical perspective, the findings may have translational relevance for prevention, diagnosis, and therapy, although the present evidence should be viewed as hypothesis-generating and requires independent validation before clinical adoption. First, for prevention, the 66 shared DEGs represent a candidate biomarker panel for exploring early detection and risk stratification in high-risk populations. Rather than serving as established screening markers, expression profiling of key inflammatory mediators such as *IL1B*, *CXCL2*, *CXCL8*, and *CCL3* in oral and intestinal tissues may help inform clinical awareness of oral–gut inflammatory comorbidity and motivate further studies on bidirectional monitoring (31). To enhance clinical utility, these candidates should undergo analytical validation (pre-analytical standardization, assay reproducibility, and cut-off optimization) and prospective verification in independent, diverse cohorts with clinically accessible specimens before consideration as screening tools.

Second, for precision diagnosis, functional characterization of myeloid subpopulations offers objective cellular metrics for disease classification and severity assessment (45). The distinct distributions of the six myeloid subsets in CD versus UC provide a cell-level framework for differential diagnosis and activity grading using single-cell assays on biopsy material. Given that scRNA-seq is not yet routine in clinical laboratories, translational proxies (e.g., immunophenotyping or targeted transcript panels for subset markers) may facilitate near-term implementation while preserving diagnostic granularity. The clustered localization of inflammatory and chemokine-related myeloid cells may also serve as dynamic indicators for monitoring progression and therapeutic response, enabling individualized care.

Third, regarding therapeutic optimization, the IL1B–chemokine regulatory axis emerges as a potentially targetable inflammatory pathway suggested by the integrated transcriptomic analyses (46, 47). In principle, strategies that attenuate IL-1 signaling and/or downstream chemokine pathways (e.g., *CXCL8/CXCR2, CCL3/CCL4–CCR* receptors) may be relevant to myeloid-driven inflammation across both periodontal and intestinal compartments (48); however, functional perturbation experiments and rigorously designed preclinical/clinical studies are required to establish causal therapeutic benefit, define efficacy–safety profiles, and determine patient-selection criteria.

Fourth, for disease management and patient education, these data underscore the tight relationship between oral and intestinal health and motivate integrated, multidisciplinary care. Dentists and gastroenterologists should coordinate bidirectional screening (oral exams for IBD patients and intestinal assessments for periodontitis patients) and co-manage care. Educating patients about the oral–gut axis—including practical guidance on periodontal hygiene as part of IBD risk mitigation and control of intestinal inflammation—may improve adherence and quality of life (7, 49).

To explore potential pathophysiological links between periodontitis and IBD, this study highlights three complementary mechanisms along the oral–gut axis. First, regarding inflammatory cascade amplification, *IL1B* emerged as a recurrent hub within the shared inflammatory program identified across datasets. Periodontal pathogens and PAMPs can activate myeloid cells in periodontal tissues to increase IL1B-related inflammatory signaling, and systemic inflammatory mediators or immune activation may contribute to intestinal immune priming in susceptible hosts (50, 51). In the IBD single-cell atlas, myeloid compartments exhibited activation of NF-κB–related inflammatory programs and coordinated upregulation of chemokine genes (*CXCL2, CXCL8, CCL3, CCL4*), consistent with an IL1B–chemokine–associated inflammatory module (52, 53). Rather than establishing a directional causal pathway, these convergent signatures support the hypothesis that heightened IL-1β–chemokine activity may represent a shared inflammatory axis that could help explain reported clinical associations and biomarker concordance between oral and intestinal inflammation.

Second, for immune microenvironment remodeling, functional polarization of myeloid subpopulations may contribute to disease activity. Under periodontal chronic inflammation, classical myeloid cells can adopt inflammatory and chemokine-producing states, reshaping local cytokine milieus (e.g., TNF-α, IL-6; neutrophil chemokines CXCL1/CXCL2) (54, 55). Systemic dissemination of inflammatory mediators and/or mobilization of activated myeloid populations has been proposed as a route of oral–gut crosstalk, and could potentially promote similar pro-inflammatory programs in intestinal tissues in susceptible hosts. Complement-related myeloid activation may further be associated with vascular activation and leukocyte recruitment through anaphylatoxins (C3a/C5a), providing a plausible molecular bridge between oral and intestinal inflammation.

Third, intercellular communication networks are extensively remodeled (56). In health, epithelial, immune, and stromal cells maintain signaling balance to preserve barrier integrity and immune homeostasis. A systemic pro-inflammatory milieu, which may occur in periodontitis and other chronic inflammatory states, could plausibly perturb this equilibrium, with the IL1B–chemokine module representing one candidate axis. Our CellChat analysis indicated that, in IBD, myeloid cells act both as major receivers and as dominant senders, relaying signals to epithelial cells, fibroblasts, and endothelial cells via *MHC-I/MHC-II, COLLAGEN, CXCL*, and other pathways. When considered alongside the periodontitis bulk-transcriptome signatures (e.g., reduced epithelial markers such as EPCAM and increased matrix-remodeling genes such as MMP7/MMP13, as well as endothelial activation markers), this communication shift supports the hypothesis that a myeloid-centered inflammatory network may be linked to multi-compartment tissue remodeling programs relevant to both oral and intestinal inflammation. However, the current data do not establish directionality, and future longitudinal and mechanistic studies will be required to test whether periodontal inflammation causally contributes to intestinal homeostatic disruption.

Despite these advances, several limitations merit consideration. First, the study largely relies on cross-sectional bulk and single-cell datasets without longitudinal sampling, precluding definitive causal inference between periodontitis and IBD. While shared DEGs and pathways were identified, association does not prove directionality or common upstream triggers. Future work should include well-designed prospective cohorts tracking incident IBD among individuals with periodontitis and longitudinal periodontal outcomes among IBD patients, to establish temporal relationships. Genetic approaches (e.g., Mendelian randomization, where suitable instruments exist) and longitudinal multi-omics could also strengthen causal inference. Second, sample representativeness may limit generalizability. Periodontitis datasets were predominantly from European/North American cohorts and the ancestry metadata for IBD single-cell samples were limited, potentially constraining applicability across populations that differ in genetics, environment, diet, and microbiota. Broader inclusion—especially from Asian and other underrepresented groups—and careful stratification by disease severity and subtype will be required to validate markers and targets. Potential confounders such as age, smoking, medications (e.g., antibiotics, immunomodulators), and disease activity should be systematically recorded and adjusted in future analyses. Third, methodological constraints may affect signal fidelity. Pseudobulk aggregation improves power but can mask single-cell heterogeneity; batch effects, capture efficiency differences, and detection bias can influence DEG calls. GSEA depends on curated pathway databases and may miss novel or disease-specific circuits, while CellChat relies on ligand–receptor priors and requires orthogonal validation. Harmonization strategies (e.g., robust batch correction, replication across platforms) and integration with proteomics/metabolomics and spatial transcriptomics would improve mechanistic resolution. Fourth, mechanistic validation remains incomplete. Although we corroborated IL-1β and CXCL2 changes in animal models, species differences and limited sample size constrain extrapolation, and not all predicted pathways were tested. Target engagement and efficacy of interventions against the IL1B–chemokine axis should be evaluated in controlled preclinical studies and early-phase trials to establish translational relevance.

## 5 Conclusion

By integrating periodontitis bulk transcriptomes with IBD single-cell data, we identified 66 shared DEGs and uncovered a convergent, myeloid-centered inflammatory program featuring an IL1B–chemokine–associated module. Cell–cell communication and myeloid subpopulation analyses implicated myeloid cells as major signaling hubs, and rat-model readouts provided supportive consistency with these computational signatures. These findings support oral–gut inflammatory crosstalk highlight an IL1β–CXCL2–associated chemokine axis as a potentially targetable myeloid signaling module, while underscoring the need for longitudinal and functional validation.

## Data Availability

The original contributions presented in the study are included in the article/**Supplementary Material**. Further inquiries can be directed to the corresponding authors.
